# Increased plasmatic NETs by-products in patients in severe obesity

**DOI:** 10.1038/s41598-019-51220-x

**Published:** 2019-10-11

**Authors:** Marco D’Abbondanza, Eva Edvige Martorelli, Maria Anastasia Ricci, Stefano De Vuono, Elisa Nulli Migliola, Cosmo Godino, Sara Corradetti, Donatella Siepi, Maria Teresa Paganelli, Norma Maugeri, Graziana Lupattelli

**Affiliations:** 1 Internal Medicine, Department of Medicine, “Santa Maria della Misericordia” Hospital, University of Perugia, Perugia, Italy; 2 Unit of General Surgery, “Santa Maria della Misericordia” Hospital, Perugia, Italy; 3 Autoimmunity and vascular inflammation Unit, San Raffaele Scientific Institute and Vita-Salute University, Milano, Italy; 4 Cardiothoracic Vascular Department, San Raffaele Scientific Institute, Milan, Italy

**Keywords:** Predictive markers, Risk factors

## Abstract

Neutrophil extracellular traps (NETs) are DNAs products involved in immune process. Obesity through a low-grade chronic inflammation determines neutrophil activation, but it is still unclear its role in NETs formation. Here we analyzed the NETs levels in healthy and morbid obese, their association with anthropometric and glyco-metabolic parameters and their changes after bariatric surgery. For this study, we enrolled 73 patients with morbid obesity (BMI ≥40 kg/m^2^ or ≥35 kg/m^2^ + comorbidity) eligible to sleeve gastrectomy. In parallel, 55 healthy subjects and 21 patients with severe coronary artery disease were studied as controls. We evaluated anthropometric parameters, peripheral blood pressure, biochemical and serum analysis at the enrollment and at twelve months after surgery. Plasmatic levels of MPO-DNA complexes were assessed by ELISA. NETs levels were higher in obese than in control group (*p* < 0.001) and correlated with the main anthropometric variable (BMI, waist, hip), glyco-metabolic variables and systolic blood pressure. NETs trend after intervention was uneven. The reduction of NETs correlated with the entity of reduction of BMI (ρ = 0.416, *p* < 0.05), visceral fat area (ρ = 0.351, *p* < 0.05), and glycemia (ρ = 0.495, *p* < 0.001). In medical history of *p*atients in whom NETs increased, we observed a higher number of thromboembolic events. Our observations indicate that severe obesity is associated with increased generation of NETs, which in turn could influence the patients’ systemic inflammatory state. Weight loss and in particular, loss of adipose tissue after bariatric surgery does not in itself correct NET’s dysregulated production. Finally, patients in whom NETs accumulation persists after surgery are probably those at the highest risk of cardiovascular events.

## Introduction

Generation of neutrophil extracellular traps (NETs) contributes to the effector function of neutrophils^1,2^. NETs are DNA filaments decorated with proteases and citrullinated histones, endowed with a bactericidal action, which is played either directly or via activation of the complement and the coagulation systems^2^. Thus NETs influence dramatically the extracellular environment and they contribute to systemic inflammation, persistent autoimmune diseases and thromboembolic events^2–6^.

Obesity increases cardiovascular risk even in the absence of other risk factors such as diabetes, dyslipidaemia, hypertension^7^ as well as rheumatic diseases^8^. Currently the extent of the generation of NETs and their role in experimental models of obesity and in obese patients is controversial^9–11^. As far as we know, only few studies have analyzed this topic. Braster *et al*.^9^ investigated the effect of NETs (by release inhibition or not) on the development of insulin resistance in a high fat diet mouse model; there was no difference in insulin resistance between treatment groups.

On the contrary, Wang *et al*.^11^ observed an effect of NETs on endothelial function (evaluated by functional studies of mesenteric arterioles) in a mouse model.

Finally, Roberts *et al*.^10^ studied a human obese patients before and after gastric band surgery observing a reduction in pro-inflammatory state and NETs production.

Bariatric surgery (metabolic surgery) has become a relevant therapeutic tool in obesity, particularly effective on obesity-associated comorbidities such as glycol-metabolic and cardiovascular dysfunctions^12^, although, as in other interventions, post-procedural weight gain is described^13^.

Here we have investigated whether byproducts of NETs generation/catabolism accumulate in the plasma of morbid obese patients, whether they correlate with the anthropometric and glyco-metabolic parameters of the patients. We have also compared NETs byproducts before and 12 months after bariatric surgery to verify whether the relative depletion of the adipose mass impacts on neutrophil function.

## Patients, Materials and Methods

### Patients

The study group consisted of 73 patients (24 males and 49 females) with morbid obesity (mean body mass index 45.5 kg/m^2^) eligible for sleeve gastrectomy were recruited. Patients with severe obesity were studied immediately before and one year after sleeve gastrectomy.

We choose to re-check these patients after twelve months because weight loss and glyco-metabolic parameters are quite stabilized in that period as documented by our previous publications^14–18^.

Inclusion criteria^19^ were BMI ≥40 kg/m^2^ or ≥35 kg/m^2^ + comorbidities (every comorbidity that should benefit of bariatric surgery such as metabolic disorders, cardiorespiratory disease, severe joint disease, obesity-related severe psychological problems) and age between 20 and 65 years.

The exclusion criteria were (**a)** all indicated for sleeve gastrectomy– liver, renal and heart failure, secondary causes of obesity (endocrine - untreated hypothyroidism, Cushing disease, etc. – pharmacologic and genetic causes), and severe psychiatric diseases (as evaluated by a consultant psychiatrist); and (**b)** patients with autoimmune (e.g. systemic lupus erythematosus, rheumatoid arthritis, small vessel vasculitis, antiphospholipid antibody syndrome and psoriasis), infectious disease or neoplasia emerging before or after surgery^19^.

We enrolled as control group 55 sex- and aged-matched healthy subjects. Moreover, we also studied a group of 21 patients with severe coronary artery disease (CAD) (with the same exclusion criteria, as described above): nine with previous acute myocardial infarction, and twelve that required angioplasty and stent implantation after coronary angiography (Table 1; Supplementary Table 1).

**Table 1:** Anthropometric and glyco-metabolic characteristic of patients and healthy controls.

	Healthy Controls			Obese Patients		
	All	Male	Female	All	Male	Female
n = 128	55	21	34	73	24	49
Age, median (range)	37 (24–75)	36 (25–65)	41 (24–75)	45.5 (20–72)	46 (22–62)	45 (20–72)
Weight (kg)	67.8 ± 10.3	74.7 ± 8.4	62.8 ± 8.7*	127.2 ± 24.8^##^	147.4 ± 23.44	117.3 ± 19.0°°
Body mass index (Kg/m^2^)	24.1 ± 2.2	23.9 ± 1.9	24.1 ± 2.5	45.5 ± 7.5^##^	48.1 ± 7.3	44.2 ± 7.6 °
Waist circumference (cm)	90.5 ± 10.4	91.7 ± 11.7	89.6 ± 9.7	133.7 ± 18.7^##^	146.7 ± 16.6	127.3 ± 16.1°°
Hip circumference (cm)	100.8 ± 8.7	100.3 ± 8.9	101.3 ± 9	143.1 ± 15.6^##^	148.9 ± 17.4	140.3 ± 14.0
Visceral fat area (cm^2^)	n.d.	n.d.	n.d.	262.8 ± 73.7	325. 9 ± 47.8	233.2 ± 64.7°°
Fat mass (kg)	n.d.	n.d.	n.d.	58.7 ± 14.2	57.7 ± 13.4	60.9 ± 16.1
Fat-free mass (kg)	n.d.	n.d.	n.d.	66.4 ± 14.7	59.5 ± 8.2	81.9 ± 14.4°°
Systolic blood pressure (mmHg)	124 ± 19	123 ± 14	124 ± 21	138.7 ± 13.9^##^	143 ± 11	137 ± 15 °
Diastolic blood pressure (mmHg)	77 ± 10	80 ± 9	75 ± 10	85.8 ± 8.8^##^	87 ± 9	85 ± 8.9
Glycemia (mg/dl)	87.3 ± 8.7	90.6 ± 7.8	85.4 ± 8.3	101.7 ± 32.1^#^	118.2 ± 48.2	93.6 ± 15.1 °
Insulinemia (μIU/mL)	9.9 ± 6.1	8.7 ± 4.9	10.8 ± 7.06	20.1 ± 14.1^##^	25.2 ± 12.4	17.8 ± 14.4 °
HOMA-IR	2.1 ± 1.4	1.9 ± 1.2	2.23 ± 1.53	4.9 ± 3.5^##^	6.5 ± 3.0	4.1 ± 3.6°°
Tryglicerides (mg/dL)	112.9 ± 45.6	108.1 ± 43.5	116.4 ± 48.9	148.5 ± 90.2	180.8 ± 75.9	132.5 ± 93.2°°
Cholesterol (mg/dL)	179.8 ± 37.4	171.7 ± 24.8	183.5 ± 45.3	195.3 ± 37.7	183.4 ± 31.7	201.3 ± 39.4
LDL-c (mg/dL)	111.5 ± 33.8	103.5 ± 20.9	117.4 ± 40.8	113.9 ± 29.7	107.1 ± 29.1	117.2 ± 29.7
HDL-c (mg/dL)	48.7 ± 14.1	43.6 ± 14.4	52.4 ± 13.2	51.6 ± 13.6	40.2 ± 7.0	57.3 ± 12.4°°
ApoA-1 (mg/dL)	n.d.	n.d.	n.d.	147.8 ± 26.2	132.6 ± 17.9	156.9 ± 26.3°°
ApoB (mg/dL)	n.d.	n.d.	n.d.	99.3 ± 25.2	99.4 ± 23.0	99.3 ± 26.8
hs-CRP (µg/mL)	0.85 ± 1.07	0.71 ± 1.2	0.94 ± 1.02	19.7 ± 19.5^##^	11.4 ± 12.4	23.9 ± 21.2 °
MPO-DNA complexes (OD)	0.11 ± 0.06	0.09 ± 0.04	0.12 ± 0.075	0.46 ± 0.16^##^	0.45 ± 0.13	0.47 ± 0.17

HOMA-IR, homeostasis model assessment for Insulin Resistance; LDL-c: low-density lipoprotein-cholesterol; HDL-c: high-density lipoprotein-cholesterol; ApoA-1: Apolipoprotein A1; ApoB: Apolipoprotein B; hs-CRP: high-sensitivity C-reactive protein. Results are expressed as mean ± SD. *P < 0.05 respect to healthy male subjects; °P < 0.05 respect to male obese patients; °°P < 0.001 respect to male obese patients; ^#^P < 0.05 respect to healthy subjects; ^##^P < 0.001 respect to healthy subjects. *P* values were determined by Mann-Whitney and chi square test, 128 subjects were analyzed. n.d. = not determined.

All patients and controls signed their consent for the study. The ethics committee of the “Santa Maria della Misericordia” Hospital University of Perugia and registered as a clinical trial as NCT03559842, while IRCCS San Raffaele Scientific Institute approved the study protocol. The study was carried out in accordance with the code of ethics of the World Medical Association for human studies (Declaration of Helsinki, 1975). All patients and controls gave their written informed consent to participate to the study.

### Anthropometric data assessment

The weight and height were measured and used to calculate body mass index (BMI). Waist and hip circumferences were measured and used to calculate the waist-hip ratio. Peripheral blood pressure was assessed with the patients in supine position by a validated device in the non-dominant arm, after 10 minutes of rest in a quiet environment. Insulin-resistance was determined using the homeostasis model assessment-insulin resistance (HOMA-IR). Visceral fat area (VFA, expressed in cm^2^) was measured at the end of a normal exhalation by ultrasonography (using a 3.5 MHz convex array probe) according to the Hirooka formula^20^. Three different distances were measured in order to apply the above-mentioned formula, as follows: VFA = −9.008 + 1.191 × [distance between the internal surface of the abdominal muscle and the splenic vein (mm)] + 0.978 [distance between the internal surface of the abdominal muscle and the posterior wall of the aorta at the umbilicus (mm)] + 3.644 [thickness of the fat layer of the posterior right renal wall (mm)]. The distance between the internal surface of abdominal muscles and the splenic vein was scanned transversely in the midline. None of the patients had dorsal or lumbar spine deformity, nor abdominal aortic aneurysm. Subcutaneous fat thickness (SFT, expressed in mm) refers to the thickness of subcutaneous fat layer as measured by ultrasonography using a 7.5-MHz linear array probe and performing a longitudinal scan 1 cm below xiphoid apophysis. Subcutaneous fat thickness was defined as the distance between the skin and external face of the rectus abdominal muscle. Bioimpedentiometry (50 kHz, amplitude 50 mA, Body Composition Analyzer TBF-410GS; Tanita, Tokyo, Japan), with electrodes applied on the plantar surface of both feet, was used to determine fat mass and free fat mass as a percentage of body weight.

### Blood sampling

Venous blood was drawn (in the morning after 13 hour fast) in vacutainers containing clot activator (to prepare serum samples) and containing EDTA (to prepare platelet free plasma). Serum and plasma were retrieved after centrifugation (3000 rpm for 10 minutes). To prepare platelet- free plasma, a second centrifugation of the obtained plasma was performed at 13,000 × g, 5 minutes at 4 °C. Platelet-free plasma retrieved were aliquoted and stored at −80 °C until quantification of NETs.

### Blood chemistry measurements

Blood sample was drawn in the morning after a 13-hour fast. Routine auto-analyzers were used to assess hematological parameters and blood chemistry including glycemia, total cholesterol (TC), high-density lipoprotein cholesterol (HDL-c), triglycerides (TG), apolipoprotein-A1 and –B and insulinemia. Low-density lipoprotein cholesterol (LDL-c) was calculated by Friedewald formula. Serum levels of hs-CRP were measured by colorimetric enzyme-linked immunosorbent assay (ELISA) following the manufacturer’s instructions (R&D Systems, Minneapolis, MN).

### NETs quantification

Plasmatic levels of MPO-DNA complexes (a bona fide marker of NET formation/catabolism) were identified using a capture ELISA as previously described^3,4,6,21^. Briefly, multiwell plates were coated with anti-human MPO mAb (capture) overnight at 4 °C, washed and blocked with BSA. After washing, human platelet-free plasma samples were placed in the coated wells with peroxidase-conjugated anti-DNA antibodies (clone MCA-33, from the cell death detection ELISA kit) following the kit instructions. Results are expressed as arbitrary units of optical density (OD).

### Statistical analysis

Analyses were performed using SPSS software for Windows (version 22.0; SPSS, Inc, Chicago, Illinois), with significance set at a 2-sided P < 0.05. Values are expressed as mean (^±^standard deviation). Kolgomorov-Smirnov test was used to determine the normal distribution of the variables. Mann-Whitney U test was used to compare the means of two unmatched groups variables (e.g. healthy and obese subjects). Differences between two paired groups before and after surgery were calculated by Wilcoxon signed-rank test. Variations of the concentration of plasmatic MPO-DNA complexes were analyzed by Mann-Whitney test and the trend in each patient recorded. Chi-Square test statistics was performed to determine if there was a significant relationship between two nominal (categorical) variables. Kruskal-Wallis test was used to compare the values of more than two different groups. Correlation coefficients were calculated with Spearman correlation rank tests, as appropriate. Eta squared analysis was used to study correlations between interval and nominal variables. The difference of different variables at time 0 and after intervention was calculated (delta, Δ).

## Results

In the present work, we analyzed 149 adults, 73 patients with severe obesity, 55 healthy controls with normal weight (Table 1) and 21 patients with coronary artery disease (Supplementary Table 1). Patients with severe obesity were studied before and one year after sleeve gastrectomy.

Among patients with severe obesity, a higher prevalence of women was observed (Table 1). Higher blood pressure values and a worse glycol-metabolic profile was observed in patients than in controls. No significantly differences in the two groups were observed in the lipid profile (cholesterol, LDL-c, triglycerides and HDL-c).

**Figure 1:**
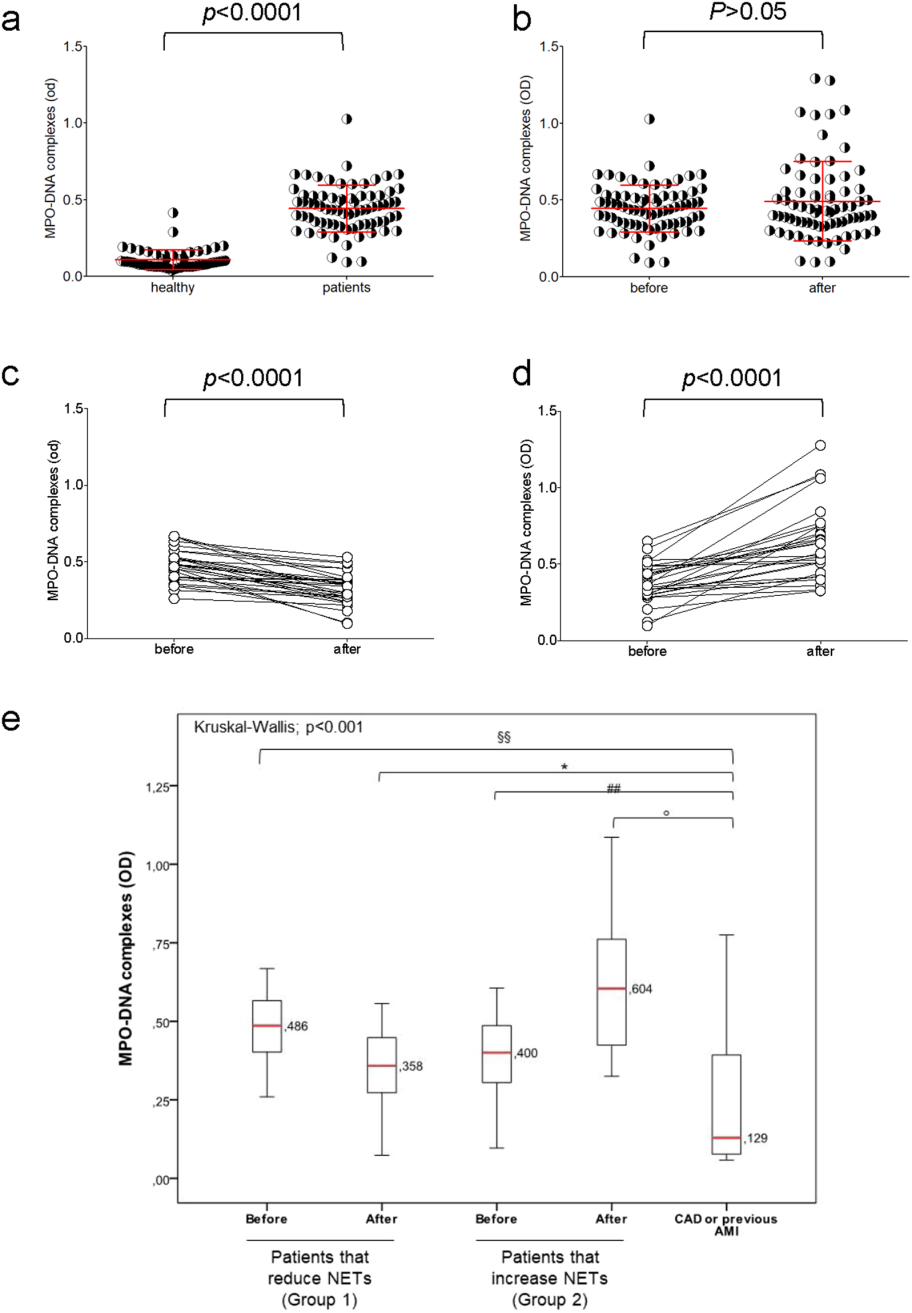
The concentration of soluble DNA-MPO complexes (a putative marker of *in vivo* NET generation/catabolism) was determine by ELISA (see methods) in platelet-free plasma samples of patients and of healthy controls **(a)** and in patients before and after gastric sleeve **(b)**. Two different groups of subjects were observed among the patients studied: those that reduce (Group 1) **(c)** and those that increased (Group 2) **(d)** the fraction of plasmatic MPO-DNA complexes after sleeve gastrectomy. **(e)** The amount of MPO-DNA complexes observed in obese patients were compared to those observed in patients with history of acute myocardial infarction (AMI) or severe coronary artery disease but without previous AMI or unstable angina. In (**a–d)**, each symbol depict the result of a single subject (patient or control). In (**a**,**b)**, red lines indicates mean ± SD. In (**e)** ^§§^P < 0.001; *P < 0.05; ^##^P ≤ 0.001; °P < 0.05; *P* values were determined by Kruskal-Wallis and Mann-Whitney Tests.

A significantly higher accumulation of DNA fragments associated to MPO was observed in patients with severe obesity than in healthy controls (0.11 ± 0.06 vs 0.46 ± 0.16; *p* < 0.001, Table 1, Fig. 1a). The concentration of MPO-DNA complexes was significantly associated to weight, body mass index, waist and hip circumferences, systolic and diastolic blood pressure and glycol-metabolic profile (Table 2). Intriguingly, the NETs-associated parameters are those that differ in obese patients compared to healthy donors (Table 1). On the contrary, lipid profile which are similar in obese and control subjects - do not correlate with the plasmatic NETs concentration (Tables 1, 2). There was no statically significant differences in NETs values between male and female obese patients (n = 73, *p* = 0.787).

**Table 2:** Correlations between the plasmatic concentration of MPO-DNA complexes and main anthropometric and glyco-metabolic parameters.

	Correlation coefficient respect to MPO-DNA complexes (ρ)	*p*
Sex (F)	0.009	0.278
Age (y)	−0.043	0.635
Weight (Kg)	0.467	<0.001
Body mass index (Kg/m^2^)	0.611	<0.001
Waist circumference (cm)	0.460	<0.001
Hip circumference (cm)	0.471	<0.001
Systolic blood pressure (mmHg)	0.205	<0.05
Diastolic blood pressure (mmHg)	0.202	0.05
Glycemia (mg/dL)	0.245	<0.05
Insulinemia (μIU/mL)	0.332	<0.001
HOMA-IR	0.371	<0.001
Tryglicerides (mg/dL)	0.187	0.08
Cholesterol (mg/dL)	0.145	0.145
LDL-c (mg/dL)	0.057	0.595
HDL-c (mg/dL)	0.027	0.800
Neutrophils (cells/μL)	0.409	<0.001
hs-CRP (µg/mL)	0.624	<0.001

HOMA-IR, homeostasis model assessment for Insulin Resistance; LDL-c: low-density lipoprotein-cholesterol; HDL-c: high-density lipoprotein-cholesterol; ApoA-1: Apolipoprotein A1; ApoB: Apolipoprotein B; hs-CRP: high-sensitivity C-reactive protein. Correlations were determined by Spearman rank test in 128 subjects (obese patients and healthy controls).

One year after sleeve gastrectomy, several parameters were significantly reduced in patients (Table 3) including the body mass index, waist and hip circumferences, glycemia, insulinemia, HOMA-IR, triglycerides, VFA. Moreover, the reduction of hs-CRP results statistically significant (19.7 ± 19.5 before vs 10.6 ± 12.6 µg/mL after surgery, *p* < 0.05). Conversely an increase in HDL-c (*p* < 0.05) was observed. In 73 patients, the plasmatic concentration of MPO-DNA complexes was determined before and after one year of sleeve gastrectomy (Fig. 1b, Table 3). Unexpectedly, there was no univocal trend and patients could be stratified into two distinct groups, one comprising patients in whom sleeve gastrectomy is associated with a decrease in the accumulation of NET by-products (Group 1, Fig. 1c) and one in which the latter is not affected or it actually increases (Group 2, Fig. 1d). This suggests that weight loss per se is not sufficient to modify the activation state of neutrophils. Of interest, there was no detectable difference in the various parameters analyzed that could be used to predict whether bariatric surgery results influences or not NET accumulation. Before and after surgery of MPO-DNA complexes as well as all other anthropometric or metabolic parameters did not differ in the two groups (Table 4). However, a significant difference is clearly detectable in terms of the fraction of patients with a history of cardiovascular events, including strokes or thromboembolism, which was significantly higher in the group in which NET production did not abate after surgery (Table 5). Surprisingly, the post-surgery values of MPO-DNA complexes in Group 2 results higher than those observed in patients with history of acute myocardial infarction or patients with severe coronary atherosclerosis (p < 0.001, Fig. 1, panel e).

**Table 3:** Variation of anthropometric and glyco-metabolic parameters after sleeve gastrectomy.

n = 73	Before	After	*p*
Weight (kg)	127.2 ± 24.8	96.3 ± 21.6	<0.001
Body mass index (Kg/m^2^)	45.5 ± 7.5	33.9 ± 6.3	<0.001
Waist circumference (cm)	133.7 ± 18.7	108.4 ± 15.7	<0.001
Hip circumference (cm)	143.1 ± 15.6	118.4 ± 14.6	<0.001
Visceral fat area (cm^2^)	262.8 ± 73.7	159.6 ± 61.2	<0.001
Fat mass (kg)	58.6 ± 14.3	34.1 ± 10.9	<0.001
Fat-free mass (kg)	66.2 ± 14.7	59.3 ± 11.7	<0.001
Glycemia (mg/dL)	101.7 ± 32.1	85.1 ± 21.4	<0.001
Insulinemia (μIU/mL)	20.1 ± 14.1	7.9 ± 4.9	<0.001
HOMA-IR	4.9 ± 3.5	1.7 ± 1.2	<0.001
Tryglicerides (mg/dL)	148.5 ± 90.2	106.2 ± 49.6	<0.001
Cholesterol (mg/dL)	195.3 ± 37.7	191.7 ± 39.1	0.292
LDL-c (mg/dL)	113.9 ± 29.7	116.5 ± 31.6	0.452
HDL-c (mg/dL)	51.6 ± 13.6	53.8 ± 12.5	<0.05
ApoA-1 (mg/dL)	147.8 ± 26.2	159.0 ± 27.6	<0.001
ApoB (mg/dL)	99.3 ± 25.2	92.8 ± 23.0	<0.05
hs-CRP (µg/mL)	19.7 ± 19.5	10.6 ± 12.6	<0.05
MPO-DNA complexes (OD)	0.46 ± 0.16	0.50 ± 0.26	0.815

HOMA-IR, homeostasis model assessment for Insulin Resistance; LDL-c: low-density lipoprotein-cholesterol; HDL-c: high-density lipoprotein-cholesterol; ApoA-1: Apolipoprotein A1; ApoB: Apolipoprotein B; hs-CRP: high-sensitivity C-reactive protein. Results are expressed as mean ± SD. *P* values were determined by Wilcoxon Test, 73 obese patients were analyzed.

**Table 4:** Differences between the groups of patients that reduce and those that increase the plasma concentration of NETs byproducts in terms of basal and after surgery values of anthropometric and glyco-metabolic parameters.

	Modification of MPO-DNA complexes after sleeve gastrectomy					
	Basal values			Values after surgery		
	Reduction	Increment	*p*	Reduction	Increment	*p*
n	40	33	—	40	33	—
Males/females (n)	13/27	11/22	0.940	13/27	11/22	0.940
Age median, yrs (range)	43.65 (20–62)	43.6 (20–67)	0.929	43.65 (20–62)	43.6 (20–67)	0.929
Weight (kg)	128.11 ± 27.9	126.1 ± 20.9	0.851	96.4 ± 22.4	96.2 ± 20.8	0.825
Body mass index (Kg/m^2^)	46.4 ± 8.6	44.3 ± 6.3	0.390	34.6 ± 6.7	33.2 ± 5.8	0.287
Waist circumference (cm)	132.9 ± 19.2	134.6 ± 18.1	0.947	107.9 ± 16.1	109.1 ± 15.5	0.839
Hip circumference (cm)	144.1 ± 16.2	141.9 ± 14.9	0.785	119.2 ± 14.9	117.2 ± 14.4	0.419
Visceral fat area (cm^2^)	260.3 ± 75.5	266.0 ± 72.5	0.825	163.8 ± 63.2	153.9 ± 59.1	0.429
Fat mass (kg)	60.1 ± 16.1	57.1 ± 11.8	0.413	34.7 ± 10.7	33.7 ± 11.1	0.636
Fat-free mass (kg)	64.4 ± 13.9	68.9 ± 15.4	0.271	59.1 ± 12.9	60.7 ± 12.6	0.678
Systolic blood pressure (mmHg)	141.2 ± 14.7	135.6 ± 12.3	0.093	125.4 ± 13.8	123 ± 14.0	0.693
Diastolic blood pressure (mmHg)	86.8 ± 9.5	84.6 ± 7.9	0.280	78.3 ± 9.3	76.4 ± 10.4	0.487
Glycaemia (mg/dL)	96.9 ± 18.9	107.5 ± 42.7	0.499	85 ± 19	85.4 ± 24.6	0.710
Insulinemia (μIU/mL)	21.2 ± 16.2	18.7 ± 11.1	0.831	7.6 ± 5.6	8.5 ± 4.2	0.165
HOMA-IR	5.1 ± 4.1	4.6 ± 2.8	0.948	1.6 ± 1.2	1.8 ± 1.1	0.269
Tryglicerides (mg/dL)	154.9 ± 106.7	140.7 ± 64.9	0.982	106.5 ± 54.8	105.8 ± 43.2	0.723
Cholesterol (mg/dL)	202.5 ± 41.9	186.3 ± 30.1	0.168	193.3 ± 39.5	189.7 ± 39.0	0.954
LDL-c (mg/dL)	118.4 ± 32.4	108.1 ± 25.3	0.284	117.1 ± 29.1	115.9 ± 34.9	0.883
HDL-c (mg/dL)	54.1 ± 14.7	48.4 ± 11.4	0.122	54.9 ± 12.0	52.6 ± 13.1	0.378
ApoA-1 (mg/dL)	153.4 ± 26.5	141.9 ± 25.0	0.121	156.4 ± 27.0	161.9 ± 28.4	0.283
ApoB (mg/dL)	102.6 ± 25.4	95.9 ± 24.9	0.330	89.6 ± 16.8	96.4 ± 28.2	0.242
Neutrophils (x10^3^/μL)	4.7 ± 1.5	4.9 ± 1.7	0.191	4.0 ± 2.5	3.9 ± 1.7	0.542
hs-CRP (µg/mL)	15.6 ± 16.7	24.2 ± 21.7	0.114	12.7 ± 15.2	8.6 ± 9.9	0.320

HOMA-IR, homeostasis model assessment for Insulin Resistance; LDL-c: low-density lipoprotein-cholesterol; HDL-c: high-density lipoprotein-cholesterol; ApoA-1: Apolipoprotein A1; ApoB: Apolipoprotein B; hs-CRP: high-sensitivity C-reactive protein. Results are expressed as mean ± SD. *P* values were determined by Mann-Whitney and chi square test, 73 obese patients were analyzed.

**Table 5:** Main differences between the groups of patients that reduce and those that increase the plasma concentration of NETs byproducts after sleeve gastrectomy.

	Patients that reduce MPO-DNA concentration after sleeve gastrectomy	Patients that increase MPO-DNA concentration after sleeve gastrectomy	*P*
N	40	33	
MPO-DNA complexes before treatment (OD)	0.5 ± 0.13	0.42 ± 0.18	<0.05
MPO-DNA complexes after treatment (OD)	0.36 ± 0.14	0.66 ± 0.27	<0.001
MPO-DNA ratio (before/after)	1.63 ± 1.3	0.67 ± 0.22	<0.001
Age median yrs (range)	43.6 ± 11	43.6 ± 12.5	0.929
Males/females (n)	13/27	11/22	0.940
Smoke (n)	10	9	0.928
Diabetes mellitus (n)	4	5	0.505
Coronary heart disease (n)	1	2	0.446
Stroke (n)	0	2	0.114
History of thromboembolic events (n)	0	4	<0.05
Hypertension (n)	15	12	0.92
Hypercholesterolemia (n)	0	1	0.653
Thyroid disease (n)	6	5	0.516
COPD (n)	2	1	0.673
Body mass index variation (before-after, Kg/m^2^)	11.8 ± 5.1	11.2 ± 3.5	0.678
Weight variation (before-after, kg)	31.7 ± 13.8	29.9 ± 15.3	0.670
Subcutaneous fat variation (before-after, mm)	12.8 ± 9.3	8.3 ± 6.6	0.056
Triglycerides variation (before-after, mg/dL)	46.0 ± 72.51	35.7 ± 68.2	0.878

COPD = chronic obstructive pulmonary disease. Results are expressed as mean ± SD. *P* values were determined by Mann-Whitney and chi square test, 73 obese patients were analyzed.

Of interest, we found a statistically significant direct correlation between the entity of reduction of NET byproduct accumulation after surgery (delta NETs, ΔNETs) and reduction of weight (ρ = 0.345, *p* < 0.05), of BMI (ρ = 0.416, *p* < 0.05), of VFA (ρ = 0.351, *p* < 0.05), and of glycemia (ρ = 0.495, *p* < 0.001) (Table 6). Patients in which NET accumulation persisted after surgery also experienced similar reduction of weight, BMI, VFA and metabolic parameters, which in this group were not associated to variations in NET concentration. This suggests that NET production (and therefore neutrophil activation state) is not per se a consequence of the presence of fat.

**Table 6:** Correlation between the entity of reduction of MPO-DNA complexes and main anthropometric and glyco-metabolic parameters after sleeve gastrectomy.

Variation of	Correlation coefficient respect to MPO-DNA complexes (ρ)	*p*
Weight (Kg)	0.345	<0.05
Body mass index (Kg/m^2^)	0.416	<0.05
Waist circumference (cm)	0.245	0.17
Hip circumference (cm)	0.271	0.156
Visceral fat area (cm^2^)	0.351	<0.05
Fat mass (kg)	0.227	0.072
Fat-free mass (kg)	−0.095	0.455
Glycemia (mg/dL)	0.495	<0.001
Insulinemia (μIU/mL)	0.136	0.473
HOMA-IR	0.212	0.260
Tryglicerides (mg/dL)	−0.045	0.806
Cholesterol (mg/dL)	−0.146	0.418
LDL-c (mg/dL)	−0.09	0.635
HDL-c (mg/dL)	0.097	0.605
ApoA-1 (mg/dL)	−0.379	0.082
ApoB (mg/dL)	0.047	0.834
hs-CRP (µg/mL)	−0.149	0.497

HOMA-IR, homeostasis model assessment for Insulin Resistance; LDL-c: low-density lipoprotein-cholesterol; HDL-c: high-density lipoprotein-cholesterol; ApoA-1: Apolipoprotein A1; ApoB: Apolipoprotein B; hs-CRP: high-sensitivity C-reactive protein. Correlations were determined by Spearman rank test in 73 obese patients.

In order to deeper analyze the role of systemic inflammation we considered two more parameters, neutrophils blood count and high sensitivity C reactive protein. Obese patients have neutrophil count higher than controls (p < 0.001, Table 7); and neutrophil counts decrease after surgery (p < 0.001, Table 7). Nevertheless, no differences in neutrophil count were observed between the group of patients that increased or reduced the concentration of plasmatic NETs byproducts after surgery (Table 4).

**Table 7:** Analysis of any association between general blood values and blood count and NETs (MPO-DNA complexes). WBC = white blood cells. 7a: P values were determined by Mann-Whitney; 7a: Correlations were determined by Spearman rank test.

A: General blood values and blood count of patients and healthy controls.						
	Healthy Controls	Obese Patients	*p*	Before surgery	After surgery	*p*
N	55	73	—	73	73	
Hematocrit (%)	42.5 ± 4.0	41.7 ± 3.7	0.238	41.7 ± 3.7	41.6 ± 5.9	0.542
WBC (x10^3^/μL)	6.2 ± 1.3	7.7 ± 2.2	<0.001	7.9 ± 1.8	7.0 ± 2.1	<0.001
Neutrophils (x10^3^/μL)	3.5 ± 1.2	4.9 ± 1.6	<0.001	4.9 ± 1.6	4.2 ± 1.9	<0.001
Platelets (x10^3^/μL)	235 ± 50	260 ± 63	<0.05	260 ± 63	245 ± 64	<0.05
**B: Correlations between the plasmatic concentration of MPO-DNA complexes and general blood values and blood count of the patients before sleeve gastrectomy**						
**Patients with severe obesity (n = 73)**	**Correlation coefficient respect to MPO-DNA complexes (ρ) before surgery**		***p***	**Correlation coefficient respect to MPO-DNA complexes (ρ) after surgery**		***p***
Hematocrit (%)	−0.034		0.779	0.217		0.076
WBC (x10^3^/μL)	0.102		0.392	0.077		0.517
Neutrophils (x10^3^/μL)	0.03		0.802	0.055		0.643
Platelets (x10^3^/μL)	0.219		0.065	0.044		0.719

Finally, we clustered obese patients in low and high levels of hs-CRP. No significant difference was found in NETs concentration between groups at high or low level hs-CRP (Table 8).

**Table 8:** NETs levels (before and after surgery) on the basis of median hs-CRP value.

Median of hs-CRP values assessed in obese patients	MPO-DNA complexes (OD)	*P*
**Before surgery**		
≤13.22 µg/mL	0.48 ± 0.16	0.539
>13.22 µg/mL	0.49 ± 0.17	
**After surgery**		
≤5.1 µg/mL	0.59 ± 0.32	0.356
>5.1 µg/mL	0.48 ± 0.22	

Obese patients were clustered taking into account the median hs-CRP values before (13.22 µg/mL) and after (5.1 µg/mL) sleeve gastrectomy, respectively. P values were determined by Kruskal-Wallis test in 73 obese patients.

## Discussion

This study provides three main new information. Firstly, severe obesity is associated with increased neutrophil activation and specifically with an increased generation of NETs, which in turn could influence the patients’ systemic inflammatory state. Secondly, weight loss and in particular loss of adipose tissue after bariatric surgery does not in itself correct NET’s dysregulated production. Third, patients in whom NET accumulation persists after surgery are probably those at the highest risk of cardiovascular events.

The accumulation of NETs in obese patients is not per se surprising. Obesity is well-known as an inflammatory condition, possibly because of the accumulation of innate immune cells that on the one hand impact on the sensitivity to insulin and on the other interfere with the function of adipocytes^22^. Neutrophils directly infiltrate adipose tissue and the vessel wall of various organs in experimental obesity^23^ while they are functionally activated, as evaluated by phagocytosis, oxidative burst, and release of granular enzymes in at least some obese subjects^24,25^. The extent of human neutrophils in circulation predicts cardiovascular events even if the mechanisms underlying neutrophil activation have not yet been elucidated^26,27^. Less information is directly available on NET generation, even if it is known that both insulin and hyperglycaemia, which are hallmarks of severe obesity (see Table 1) have been suggested to facilitate neutrophil activation^28^. Our data however do not support a direct link between hyperinsulinemia, hyperglycaemia and increased NET generation, since the bariatric surgery, which improves or corrects these metabolic features in virtually all patients, does not necessarily quench NET accumulation in the patients’ plasma. On the other hand our data well agree with the demonstration obtained in experimental models that inhibition of NET generation does not per se influence the accumulation of adipose tissue^29^. Other signals are likely to be involved in supporting NET generation that are not necessarily modified early after surgery.

Endothelial activation is a promising candidate. Neutrophils interact effectively both with endothelial cells and activated platelets at sites of inflammation and previous studies have correlated soluble markers reflecting endothelial activation in patients and atherosclerosis in experimental animals with NET generation^21^. Further studies are necessary to verify the association of increased amounts of NETs in obese subjects, with atherosclerosis and increased cardiovascular risks, thrombosis in particular^27,30,31^. Indeed, NETs were associated with systemic vascular inflammation and endothelial damage^26,30,32^. Of interest, neutrophils purified from the peripheral blood of obese patients appear to have an impaired capacity to generate NETs *in vitro*, possibly reflecting previous functional exhaustion *in vivo*^30^. The actual site in which neutrophils generate NETs in obese patients, whose byproducts accumulate in the peripheral blood, remain to be identified.

Finally our data indicate a dramatic heterogeneity in the obese population in terms of the regulation of NET generation. A fraction of patients indeed respond nicely to bariatric surgery, with a substantial correction of the accumulation of NETs byproducts (group 1). It is tempting to speculate that neutrophils infiltrating the adipose visceral tissues are the main source of NETs in these subjects (correlations). Conversely, a distinct group of patients fail to respond in terms on NET accumulation to surgery, and this group comprises subjects with an higher incidence of cardiovascular events. The possible role of non-fat associated stimuli supporting neutrophil activation and in particular of the reciprocal activation of neutrophils, platelets and endothelial cells^33,34^ in this group of patients need to be experimentally verified.

## Supplementary information

Anthropometric characteristics, general blood values and NETs non obese patients with severe coronary artery disease
