# Supplementary material for: Increased plasmatic NETs by-products in patients in severe obesity
Source: Sci Rep. 2019 Oct 11;9:14678. doi: 10.1038/s41598-019-51220-x (PMC6789039; doi:10.1038/s41598-019-51220-x)
Supplement: Supplementary file 1 — Anthropometric characteristics, general blood values and NETs non obese patients with severe coronary artery disease [file 41598_2019_51220_MOESM1_ESM.pdf]

## SUPPLEMENTARY MATERIAL

# Increased plasmatic NETs by-products in patients in severe obesity

*Marco D'Abbondanza[1], Eva Edvige Martorelli[1], Maria Anastasia Ricci[1], Stefano De Vuono[1], Elisa Nulli Migliola[1], Cosmo Godino[4], Sara Corradetti[4], Donatella Siepi[1], Maria Teresa Paganelli[2], Norma Maugeri[3*] and Graziana Lupattelli[1]*

[1] Internal Medicine, Department of Medicine, "Santa Maria della Misericordia" Hospital, University of Perugia-Italy
[2] Unit of General Surgery, "Santa Maria della Misericordia" Hospital, Perugia -Italy
[3] Autoimmunity and vascular inflammation Unit. San Raffaele Scientific Institute and Vita-Salute University, Milano
[4] Cardiothoracic Vascular Department, San Raffaele Scientific Institute, Milan, Italy.

**Running Title:** NETs in severe obesity.

**Key words:** *NETs; Neutrophil Extracellular Traps; obesity; sleeve gastrectomy.*

# Supplementary Figures

A)

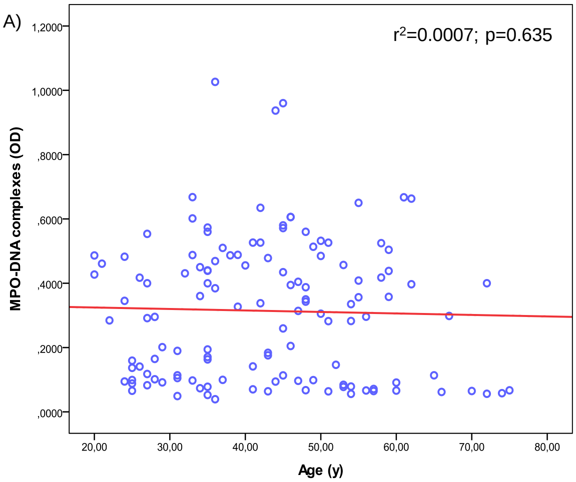


B)

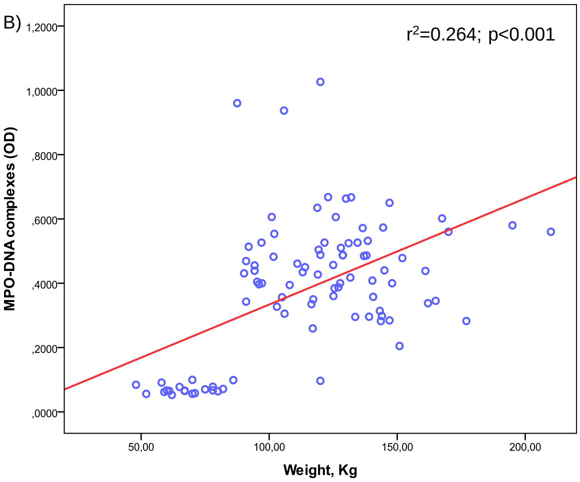


C)

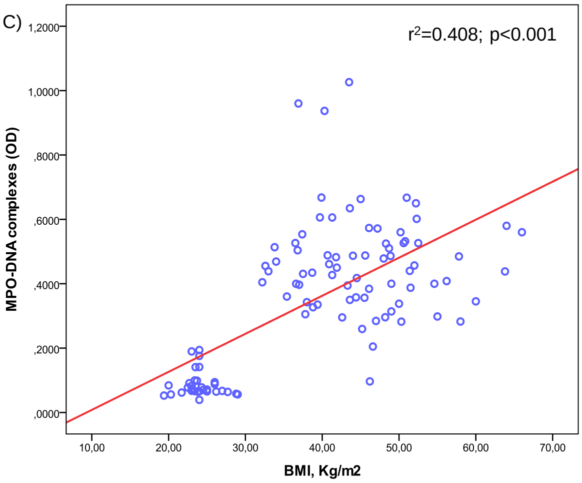


D)

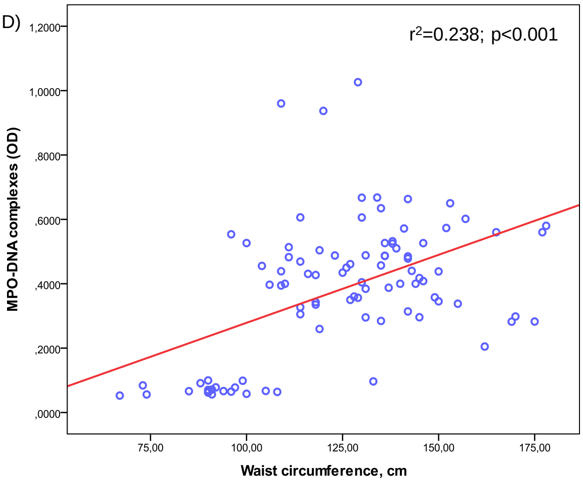


E)

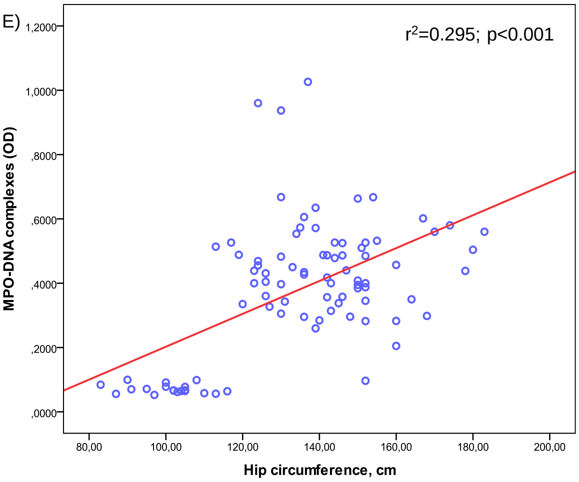


F)

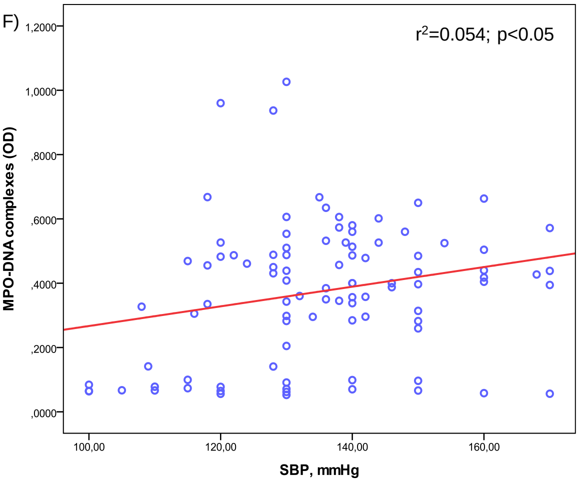

# SREP-18-49809A_R1
# Supplementary Figures

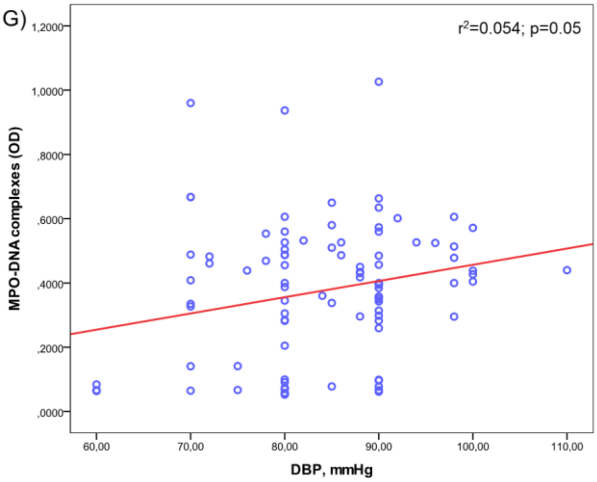


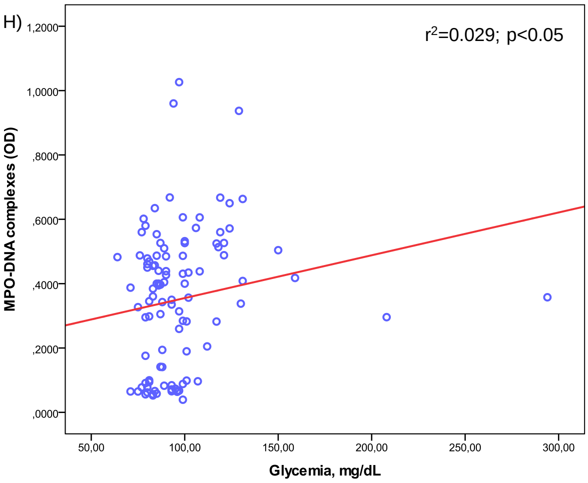


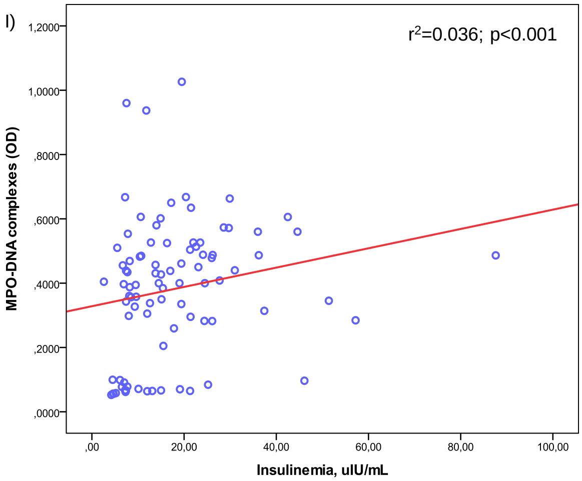


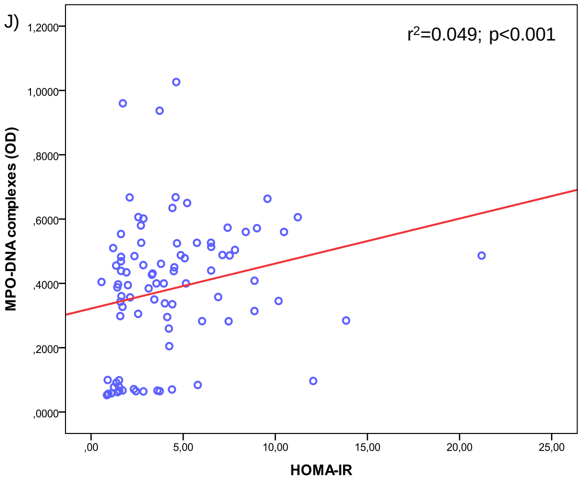


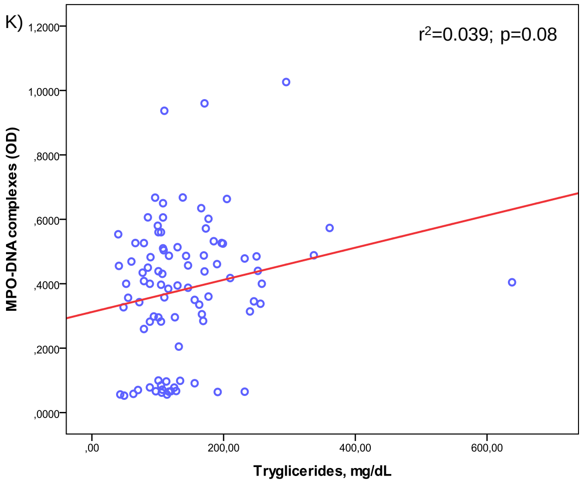


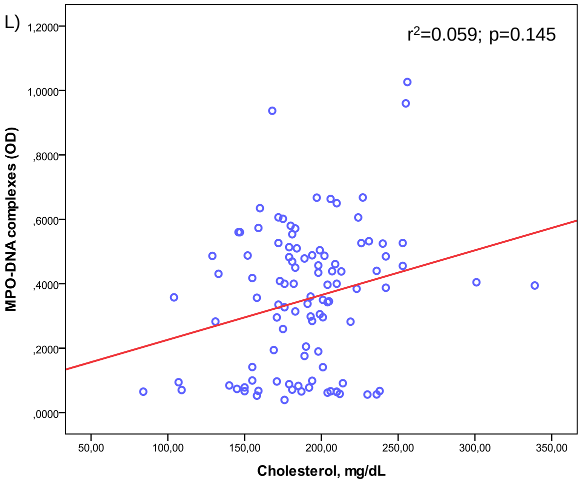

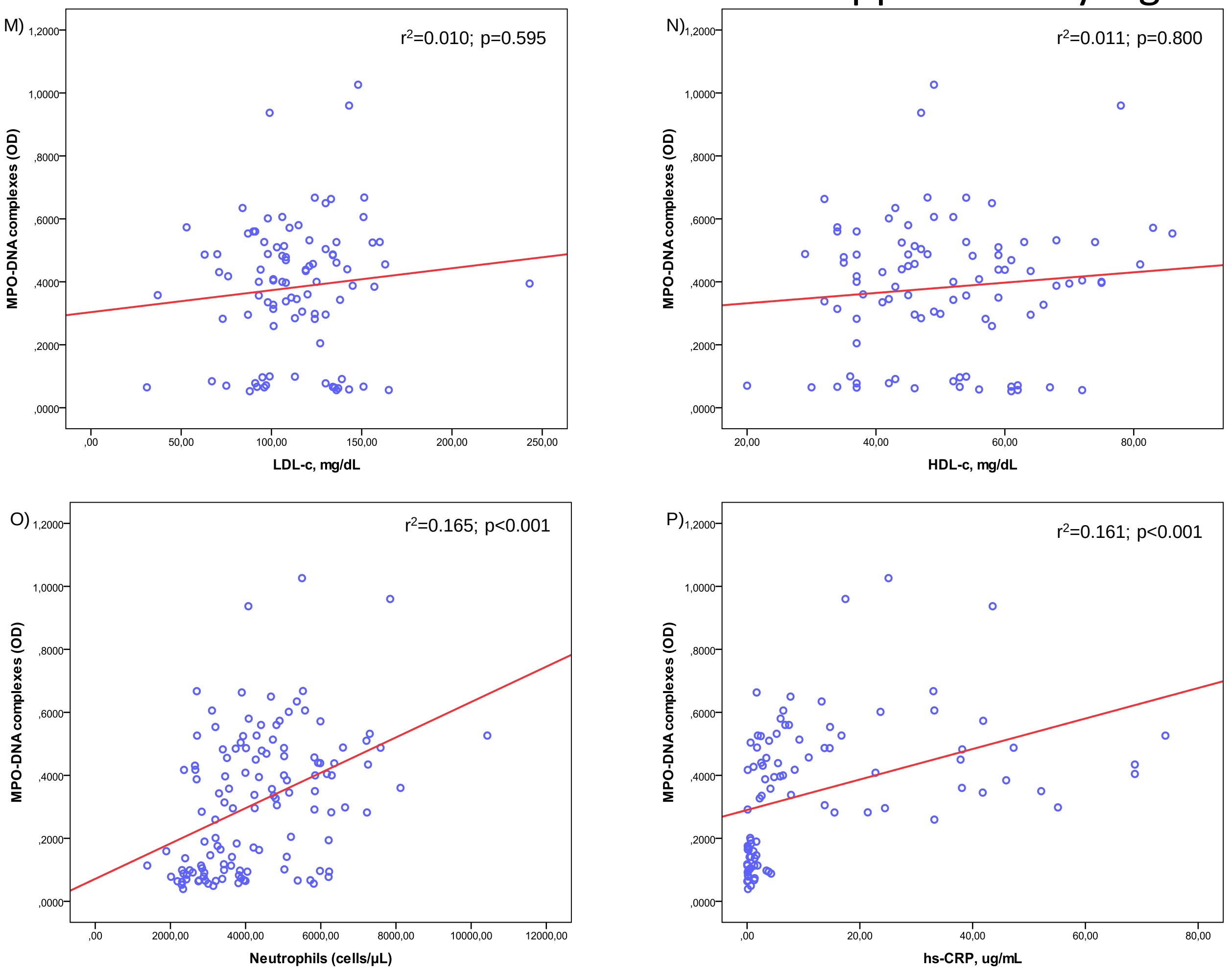


Supplementary figure 1 A-P) - Correlations' graphs between the plasmatic concentration of MPO-DNA complexes and main anthropometric and glyco-metabolic parameters.

A) Age; B) Weight; C) Body mass index; D) Waist circumference; E) Hip circumference; F) Systolic blood pressure; G) Diastolic blood pressure; H) Glycemia; I) Insulinemia; J) HOMA-IR (homeostasis model assessment for Insulin Resistance); K) Tryglicerides; L) Cholesterol; M) LDL-c (low-density lipoprotein-cholesterol); N) HDL-c (high-density lipoprotein-cholesterol); O) Neutrophils; P) hs-CRP (high-sensitivity C-reactive protein).

In A-P, each symbol depict the result of a single subject.

*P* values were determined by Spearman rank test in 128 subjects (obese patients and healthy controls).

Supplementary Table 1 - Anthropometric characteristics, general blood values and NETs non obese patients with severe coronary artery disease

| | **Previous AMI** | **Severe atherosclerosis without AMI or unstable angina** |
|---|---|---|
| N | 9 | 12 |
| Age, median (range) | 67 (47-79) | 67 (50-78) |
| Sex (F) | 1 | 4 |
| Weight (kg) | 85±13 | 79±12 |
| Body mass index (Kg/m$^2$) | 29±2 | 27±3 |
| Hb (g/dL) | 15±1 | 14±1 |
| Neutrophilsx10$^3$/µL | 5±1 | 5±1 |
| Platelets Count x 10$^3$/µL | 231±40 | 219±59 |
| Cholesterol (mg/dL) | 135±27 | 163±27 |
| LDL-c (mg/dL) | 77±27 | 94±25 |
| HDL-c (mg/dL) | 44±11 | 50±12 |
| MPO-DNA complexes (OD) | 0.22±0.24 | 0.26±0.24 |

AMI acute myocardial infarction; Hb: haemoglobin. LDL-c: low-density lipoprotein cholesterol; HDL-c: high-density lipoprotein-cholesterol. Data are expressed as mean ±SD.
